# Resting-State Functional Connectivity in Late-Life Depression: Higher Global Connectivity and More Long Distance Connections

**DOI:** 10.3389/fpsyt.2012.00116

**Published:** 2013-01-09

**Authors:** Iwo Jerzy Bohr, Eva Kenny, Andrew Blamire, John T. O’Brien, Alan J. Thomas, Jonathan Richardson, Marcus Kaiser

**Affiliations:** ^1^School of Computing Science, Newcastle UniversityNewcastle upon Tyne, UK; ^2^Institute for Ageing and Health, Newcastle UniversityNewcastle upon Tyne, UK; ^3^Newcastle Magnetic Resonance Centre, Campus for Ageing and Vitality, Newcastle UniversityNewcastle upon Tyne, UK; ^4^Institute of Cellular Medicine, Newcastle UniversityNewcastle upon Tyne, UK; ^5^Institute of Neuroscience, Newcastle UniversityNewcastle upon Tyne, UK; ^6^Department of Brain and Cognitive Sciences, Seoul National UniversitySeoul, South Korea

**Keywords:** late-life depression, aging, resting-state, functional connectivity, default mode network, network analysis, graph theory, functional magnetic resonance

## Abstract

Functional magnetic resonance imaging recordings in the resting-state (RS) from the human brain are characterized by spontaneous low-frequency fluctuations in the blood oxygenation level dependent signal that reveal functional connectivity (FC) via their spatial synchronicity. This RS study applied network analysis to compare FC between late-life depression (LLD) patients and control subjects. Raw cross-correlation matrices (CM) for LLD were characterized by higher FC. We analyzed the small-world (SW) and modular organization of these networks consisting of 110 nodes each as well as the connectivity patterns of individual nodes of the basal ganglia. Topological network measures showed no significant differences between groups. The composition of top hubs was similar between LLD and control subjects, however in the LLD group posterior medial-parietal regions were more highly connected compared to controls. In LLD, a number of brain regions showed connections with more distant neighbors leading to an increase of the average Euclidean distance between connected regions compared to controls. In addition, right caudate nucleus connectivity was more diffuse in LLD. In summary, LLD was associated with overall increased FC strength and changes in the average distance between connected nodes, but did not lead to global changes in SW or modular organization.

## Introduction

Late-life depression (LLD) is a common psychiatric disorder that typically occurs after 60 years of age. Prevalence rates can range from 1 to 4% for major and up to 13% for minor depression. Whereas volume reductions in cortical- and subcortical regions can be found, it is unclear what the consequences for cognitive functions may be. In this study, resting-state (RS) functional magnetic resonance imaging (rs-fMRI) is used to observe functional connectivity (FC) indicating correlated activity patterns in different parts of the brain (Fox and Raichle, 2007; Auer, 2008). In rs-fMRI, spontaneous low-frequency fluctuations (SLFs, 0.01–0.1 Hz) occur in the blood oxygenation level dependent (BOLD) signal in globally distributed brain areas, which form functionally related networks, termed RS networks (RSNs; Fox and Raichle, 2007; Auer, 2008; van den Heuvel and Hulshoff Pol, 2010). Default Mode Network (DMN) SLFs are negatively correlated with tasks requiring focused attention (Raichle et al., 2001; Greicius et al., 2003; Buckner et al., 2008). The DMN includes the ventral medial prefrontal cortex and the posterior cingulate cortex (PCC) also stretching to the precuneus (PC) and intraparietal lobule. Primary sensory or motor regions are absent from the DMN (Buckner et al., 2008).

There are two main approaches to investigate FC: hypothesis-driven and data-driven. Hypothesis-driven approaches involve the selection of a seed and FC is investigated with either a pre-defined brain region(s) or all other brain voxels by correlation of the SLF in the seed region with the other brain regions. In contrast, data-driven approaches are not based on any *a priori* hypothesis about the importance of specific brain areas and look into patterns emerging as a result of the analysis of the activity in the brain as a whole. Compared to a previous hypothesis-driven LLD study (Kenny et al., 2010), we here use a data-driven approach.

We apply network analysis to characterize whole brain changes in FC. Network analysis provides a range of tools for studying brain regions (treated as nodes of the network) and interactions (edges; Sporns et al., 2004; Reijneveld et al., 2007; Stam and Reijneveld, 2007; Bullmore and Bassett, 2010; Kaiser, 2011).

Brain connectivity was found to show properties of Small-World (SW) networks (Watts and Strogatz, 1998) for various techniques (fMRI, EEG, tract tracing) and various species and levels of organization (*C. elegans*, rat, cat, macaque, human). SW networks are characterized by a relatively small number of links that must be passed to “travel” between a pair of nodes. This may be expressed as the characteristic path length; *L*. SW networks also display high values of interconnectedness of neighboring nodes (high clustering coefficient, *C*). Therefore, SW properties in brain networks ensure efficient processing while reducing the total cost of wiring (Bassett and Bullmore, 2006; Kaiser and Hilgetag, 2006).

Brain networks also show a hierarchical modular organization (Bassett et al., 2010) and contain highly connected nodes or hubs (Hagmann et al., 2008). Hubs are often critical for the structural and functional integrity of a network. In many cases they play a role of “bridges” between nodes and often between clusters, thus assuring a low value of characteristic path length. For RS FC, most hubs are part of the DMN; for example, the PC or parietal and medial prefrontal cortex (Buckner et al., 2009). Hubs have been characterized by a high number of long distance connections (Achard et al., 2006) and a tendency toward an inverse relationship between Euclidean distances (EDs) and fluctuation frequency (Salvador et al., 2005). A number of diseases have an impact on FC (Buckner et al., 2008; Bassett and Bullmore, 2009), including Alzheimer’s disease (Supekar et al., 2008), schizophrenia (Liu et al., 2008), and depression (Zhang et al., 2011). It has been proposed that neurodegenerative diseases specifically target critical network components, such as hubs and sets of hubs (Buckner et al., 2009; Seeley et al., 2009); therefore, alterations in RSNs might be causes rather than consequences of these disorders.

Depression can be categorized as either major or minor based on duration, number of symptoms, and severity. Five of the core symptoms must be present for at least 2 weeks for a diagnosis of major depression to be fulfilled; one symptom must be depressed mood or loss of interest/enjoyment in everyday activities (anhedonia). The symptoms must have a significant impact on occupational and/or social functioning in order for criteria to be fulfilled (Meunier et al., 2009). LLD, typically occurring after 60 years of age, can cause great suffering in the elderly and reduce their quality of life. LLD is frequently comorbid with physical illnesses, for example it is common in patients recovering from myocardial infarction (MI; American Psychiatric Association, 1994), and when present can delay recovery and lengthen hospital stay. Compared to other diseases, there are few studies on the relationship between FC and LLD. Findings have varied with some reporting increased connectivity (Kenny et al., 2010), others increased and decreased connectivity (Yuan et al., 2008), and others decreased only (Aizenstein et al., 2009).

In this study, we measured RS FC using a data-driven analysis approach thus extending the findings from a previous hypothesis-driven study that used a seed correlation analysis approach (Kenny et al., 2010). Note that we selected a group of patients not displaying symptoms of depression at the time of the investigation since our aim was to look at the traits rather than the state of the disease (see also Materials and Methods and Discussion for more details on this matter).

## Materials and Methods

### Participants

This study involved 30 subjects: 14 with a history of major depression (LLD group) and 16 (age-matched) control individuals. Patients were recruited from consecutive referrals to Newcastle and Gateshead Old Age Psychiatry Services. All subjects were aged 65 years or older. Control participants were recruited by advertisement; none of the control subjects had past or present history of depression. A full neuropsychiatric assessment was conducted including family history of depression, previous psychiatric history, medical history, and current medication. Current depression severity was rated using the Montgomery–Åsberg Depression rating scale (MADRS; Montgomery and Asberg, 1979). Depressed subjects were required to fulfill DSM-IV criteria for a life-time diagnosis of major depressive episodes (American Psychiatric Association, 1994). Patients were assessed by senior psychiatrists in the NHS and then by a senior research psychiatrist (JR) who applied DSM criteria. All psychiatrists were MRCPsych and fully trained, equivalent of Board Certified in US. Comorbidity was assessed by physical examination, including cardiovascular and ECG, by Jonathan Richardson.

All subjects were also assessed on the Mini Mental State Examination (MMSE) to exclude the presence of dementia (Folstein et al., 1975). For all participants, the following exclusion criteria applied: dementia or MMSE < 24 (absence of dementia in referred subjects was confirmed by AV), current use of a tricyclic antidepressant, comorbid or previous drug or alcohol misuse, previous head injury, previous history of epilepsy, previous transient ischemic attack (TIA), or stroke, a carotid bruit on physical examination, MI in the previous 3 months, a depressive episode in the previous 3 months, or contraindication to MRI screening. The study was approved by the Newcastle and North Tyneside Research Ethics Committee and all subjects gave verbal and written consent.

Table 1 shows the clinical characteristics of the study subjects. Groups were comparable for gender (χ^2^ = 1.2, *df* = 1), age, and MMSE score. Mean MADRS score for LLD subjects was 7.5, indicating that most had recovered from their episode of depression by the time of scanning. Mean age at onset of depression was 49.8 years and the number of previous episodes of depression was 2.6. At the time of the study, four LLD subjects were taking antidepressants (citalopram and lofepramine), two were taking antipsychotics (flupenthixol and prochlorperazine), one was taking non-benzodiazepine hypnotic (zopiclone), and one an antiepileptic drug (carbamazepine).

**Table 1:** **Demographic and neuropsychological data of controls and late-life depression (LLD) patients**.

Demographic/neuropsychological data	Controls	LLD	*p* Value
N	16	14	
Sex (M:F)	10:6	8:6	0.27^a^
Age (years)	75.8 ± 7.8	76.6 ± 7.7	0.77^b^
MMSE	28.9 ± 1.2	28.0 ± 1.9	0.27^b^
MADRS		7.5 ± 4.7	
Age at onset of depression		49.8 ± 18.8	
No. of previous episodes of depression		2.6 ± 2.1	

*Values expressed as mean ± SD. MADRS, Montgomery–Asberg depression rating scale; MMSE, mini mental state examination*.

*^a^The *p* value was calculated using χ^2^ test*.

*^b^The *p* values were calculated using the independent-samples *t*-test*.

### Image acquisition and pre-processing

Images were acquired using a 3 T scanner (Intera Achieva, Philips Medical Systems, The Netherlands), with an eight-channel head coil. Conventional T1-weighted three-dimensional scans: magnetization-prepared rapid acquisition with gradient echo (MPRAGE) were collected for anatomical mapping. Sagittal slices were acquired of thickness = 1.2 mm, voxel size = 1.15 mm × 1.15 mm, repetition time (TR) = 9.6 ms, echo time (TE) = 4.6 ms, flip angle = 8°, SENSE factor = 2.

Subjects were instructed to lie still in the scanner, to keep their eyes closed but not to fall asleep while RS images were collected using a gradient echo echo-planar imaging (GE-EPI) sequence with the following parameters: TE = 40 ms, TR = 3000 ms, flip angle 90°, 25 contiguous axial slices of 6 mm thickness, field of view (FOV) = 260 mm × 260 mm, in-plane resolution 2 mm × 2 mm. A total of 128 volumes were collected per subject, with a total scan time of 6.4 min. As previously shown, this number of volumes is sufficient to obtain stable network features (van Wijk et al., 2010).

Images were pre-processed using FSL (Smith et al., 2004; Woolrich et al., 2009) to correct for subject motion (MCFLIRT; Jenkinson et al., 2002) and to extract the brain from non-neural tissue (BET; Smith, 2002). We also applied spatial smoothing (5 mm full width at half maximum) and high-pass temporal filtering (cut-off = 125 s; FEAT, version 5.92). To account for age-related anatomical changes, such as ventricular enlargement or gyri shrinking, anatomical scans were transformed to standard space and averaged to create a subject-specific template for registering our functional imaging data.

Anatomical T1 images were segmented into gray matter, white matter, and cerebrospinal fluid (CSF) using SPM5 (Wellcome Department of Imaging Neuroscience Group, London, UK) implemented in Matlab R2009a (Mathworks, Inc., Natick, MA, USA), and total intracranial volume was calculated from the sum of the three components. We did not find a significant difference in brain volumes between controls and the LLD group using unpaired two-sample *t*-test.

### Functional connectivity analysis workflow

**Figure 1:**
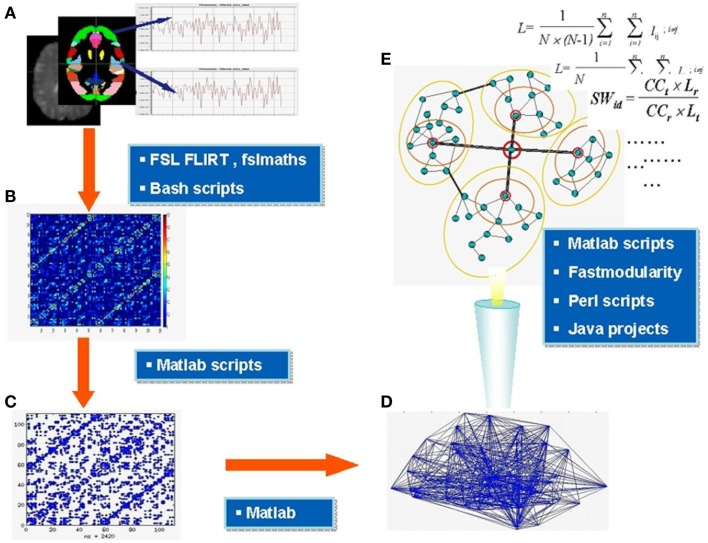
**Major steps of functional connectivity analysis**. Parcellation of the brain into areas based on the anatomical atlas and extraction of demeaned time series BOLD signal from each area **(A)**, construction of correlation matrices **(B)** thresholding and binarization of correlation matrices; generation of binary adjacency matrices **(C)** visualized in **(D)**, analysis of topology and microcircuit patterns **(E)**. In the blue boxes are the names of main software tools used at relevant stages. Section “Materials and Methods” for further details.

All major steps of the workflow are summarized in Figure 1. Parcellation was performed using FSL and was based on the Harvard-Oxford Probabilistic MRI Atlas (HOA). This involved extracting 48 cortical and seven subcortical regions (thalamus, caudate, putamen, pallidum, amygdala, nucleus accumbens, and hippocampus) from the respective parts of the atlas, thus totaling in 110 brain regions in two hemispheres. Note, that network properties relate to the number of nodes in a network (Echtermeyer et al., 2011) and we therefore chose 110 nodes to be comparable with majority of previous whole brain networks studies based on macroanatomical atlases; see for instance a recent paper indicating similar results of FC analysis using three types of macroanatomical atlases (Spoormaker et al., 2012). FLIRT was used to register structural images to functional images, averaging over each ROI for each volume, and demeaned time series for each area extracted. Using custom scripts in Matlab (Release 2009a), data from each individual were placed in one temporary matrix for each subject (*n* × *m*; *n* = number of nodes = 110, *m* = number of scans = 128), global signal removed (mean BOLD signal subtraction for all nodes), and transformed into correlation matrix (CM) representing all 110 nodes. Self-correlations, across the diagonal of CM, were disregarded.

### Network analysis

The raw CM represents weighted un-directed graphs. We observed the average correlation between all pairs of nodes (cross-correlation matrix). This procedure was applied to (a) the raw CMs, (b) CMs with negative correlation values set to zero, and (c) CMs with a percentage of top positive correlations remaining and all other correlations set to zero. The latter CMs were used to generate binary networks, setting all non-zero values to 1. For this, the 20% of top correlations (Pearson *r*-values) were considered as functionally connected nodes. Such thresholding led to equal edge densities in all subjects, which is required for comparisons of network topology. Using different edges densities, e.g., by using a constant correlation value as threshold for all subjects, would otherwise directly influence network features. In addition, we chose a 20% edge density to be in line with what would be expected from the edge density of the underlying structural connectivity which ranges from 10 to 30% (van Wijk et al., 2010). The 20% edge density led to an average correlation threshold of *r* = 0.28 which is close to the threshold in an earlier study (Kaiser, 2011).

We calculated several topological features for the thresholded binary networks (see Appendix or Achard et al., 2006; Bassett and Bullmore, 2006 for more details): first, the characteristic path length *L*, which is the average number of connections that have to be crossed to go from one node to another on the shortest-possible path. Second, the clustering coefficient *C*, that defines what proportion of neighbors (nodes which are directly connected to a node) are connected to each other. SW networks are characterized by a clustering coefficient that is much higher than for a randomly connected network while the characteristic path length is still comparable to that of a random network (Kaiser, 2011). A way to assess the extent of such a SW organization is the small-worldness σ as defined by σ = *C* *L*_r_/(*C*_r_ *L*) where *L*_r_ and *C*_r_ are the characteristic path length and clustering coefficient of a random benchmark network, respectively (Watts and Strogatz, 1998). Third, we observed the modularity *Q* that determines the degree to which a network is organized into distinct modules. In addition to topological changes, we also searched for changes in spatial organization. The three-dimensional location of a node was given by the centre of mass of a region’s coordinates in FSL. The ED between connected nodes was used as an approximation of the connection reach.

### Statistical analysis

Values for metrics of global FC are quoted as mean ± SD. Two-sample *t*-tests were performed to check for statistical differences of single measures between the two groups, with *p* < 0.05 thresholds for significance of global measures and *p* < 0.01 for node-wise analysis (corrected for multiple comparisons; number of nodes: 110). All correlations were tested with Pearson coefficient (*r*) and with *t*-test (*n* − 2 degree of freedom; *n* = number of rows in a correlation matrix) for significance. To correct for multiple comparisons in the case of node-wise analysis, we used non-parametric permutation tests (Humphries and Gurney, 2008; 5,000 iterations) with a False Discovery Rate (FDR) of 5% (implemented by Dr. Cheol Han in a Matlab script). Analysis was performed using SPSS (version 15.0.1) and Matlab.

## Results

### Global network

Late-life depression showed a higher association at a global level as measured by the cross-correlations *r*, averaged across all subjects in each group (*p* = 0.037): *r*_av_ = 0.006483 ± 0.010662 vs. *r*_av_ = 0.000411 ± 0.00482 for patients and controls, respectively. There was no difference between groups after setting negative correlations to zero.

Global network measures for binary networks (*L*_av_, *C*_av_, γ, λ, σ, and *Q*) yielded very similar values for both groups (Table 2). The values for average characteristic path length *L*_av_ were similar for controls and LLD participants (2.20 ± 0.14 and 2.20 ± 0.19, respectively), as was the value for average clustering coefficient *C*_av_ (0.58 ± 0.05 in both groups). The values of *L* and *C* suggest a SW architecture of the FC networks. This is confirmed by high values of small-worldness σ (2.30 ± 0.07 and 2.27 ± 0.13, respectively) and consistent with the ratio of path lengths γ (2.78 ± 0.25 and 2.80 ± 0.21) and of clustering coefficients λ (1.20 ± 0.08 and 1.23 ± 0.11) between FC and benchmark random networks with the same number of nodes and edges. These findings indicate that the LLD group preserved SW and modular characteristics despite the mental changes caused by depression. Interestingly, there was no correlation between clustering coefficient *C* and modularity *Q* in LLD whereas these two measures of modular organization strongly correlate with each other within controls (*r* = 0.6; *p* < 0.05).

**Table 2:** **Summary of global aggregate measures in the two groups (means ± SD)**.

	Controls	LLD
Grand mean for row cross-correlation matrices	0.000411 ± 0.0048	0.00648 ± 0.011*
Grand mean for thresholded cross-correlation matrices	0.454229 ± 0.05358	0.465225 ± 0.071135
Characteristic path length (L)	2.20 ± 0.14	2.20 ± 0.19
Clustering coefficient (CC)	0.58 ± 0.05	0.58 ± 0.05
γ	2.78 ± 0.25	2.80 ± 0.21
λ	1.20 ± 0.08	1.23 ± 0.11
Small-world index (σ)	2.30 ± 0.07	2.27 ± 0.13
Modularity (*Q*)	0.39 ± 0.04	0.37 ± 0.03

*Significantly higher (*p* < 0.05, *t*-test).

### Local regions

**Figure 2:**
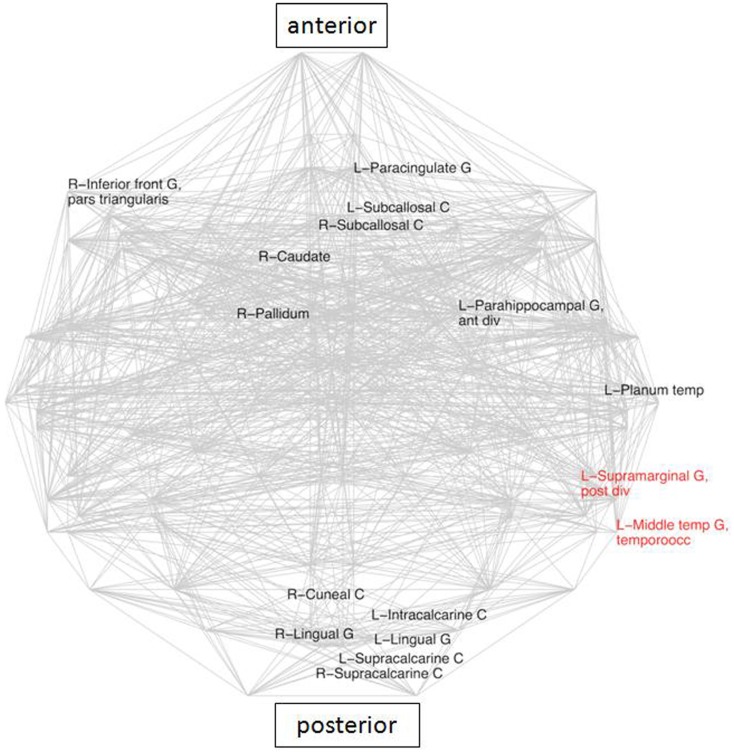
**Areas with significantly different average Euclidean distances to its neighbors (inter-group differences), superimposed on the whole brain connectivity projected onto one axial plane, averaged for all subjects in each group (pale gray lines), FDR: 5% corrected**. LLD-related increases in black, decreases in red. R\L, right\left hemisphere; front, frontal; G, gyrus; inf, inferior; occ, occipital; temp, temporal.

Topological measures, when applied to each node separately, did not yield significant inter-group differences (*L_i_*, *C_i_*, σ, and *k*). In contrast, mean EDs of neighbors changed for several nodes (Figure 2). The average distance between connected nodes in LLD patients was significantly higher than in the control group for 14 regions and significantly lower than in the control group for two brain areas in the left hemisphere: Middle Temporal Gyrus, and Supramarginal Gyrus (SG; all significant differences at 5% FDR).

Composition of the top 15 hubs (Table 3) did not yield significant differences. In addition there was a great deal of inter-individual variance in the two groups as far as composition of this core is concerned. The hub that occurred most consistently (60% in both groups) within the core was the posterior supramarginal gyrus (PSG). In controls the second most frequently occurring hub was a frontal area: middle frontal gyrus (MFG), whereas in LLD it was an anterior division of the SG (53%) coming in at third position in frequency ranking (33%), slightly ahead of the PCC PCC (27%), which was less frequent in controls (20%). Therefore a tendency toward more medial-parietal areas as the most frequent hubs in LLD was observed. This was in contrast to controls in which frontal and temporal areas seemed to dominate.

**Table 3:** **List of 15 top hubs for controls and LLD group**.

Controls		LLD	
Area name	*k*	Area name	*k*
R-paracingulate G	38	R-paracingulate G	46
R-supramarginal G, posterior division	37	L-supramarginal G, ant. division	43
L-middle frontal G	36	L-paracingulate G	42
L-paracingulate G	36	L-central opercular C	42
R-lateral occipital C, superior division	36	L-precentral G	41
L-cingulate G, ant. division	34	R-pariet. operculum	41
L-central opercular C	34	L-supramarginal G, posterior division	39
R-angular G	33	L-cingulate G, ant. division	39
L-pariet. operculum	33	L-pariet. operculum	39
L-supramarginal G, posterior division	32	R-lateral occipital C, superior division	39
L-putamen	32	R-supramarginal G, posterior division	38
R-frontal pole	32	R-angular G	38
R-juxtapositional lobule C (formerly supplementary motor C)	32	R-cingulate G, ant. division	38
R-cingulate G, ant. division	32	R-precuneus C	37
L-insular C	31	L-lateral occipital C, superior division	36

*L, left; R, right; *k*, degree centrality values; C, cortex; G, gyrus; ant., anterior; pariet., parietal*.

### Local circuits

**Figure 3:**
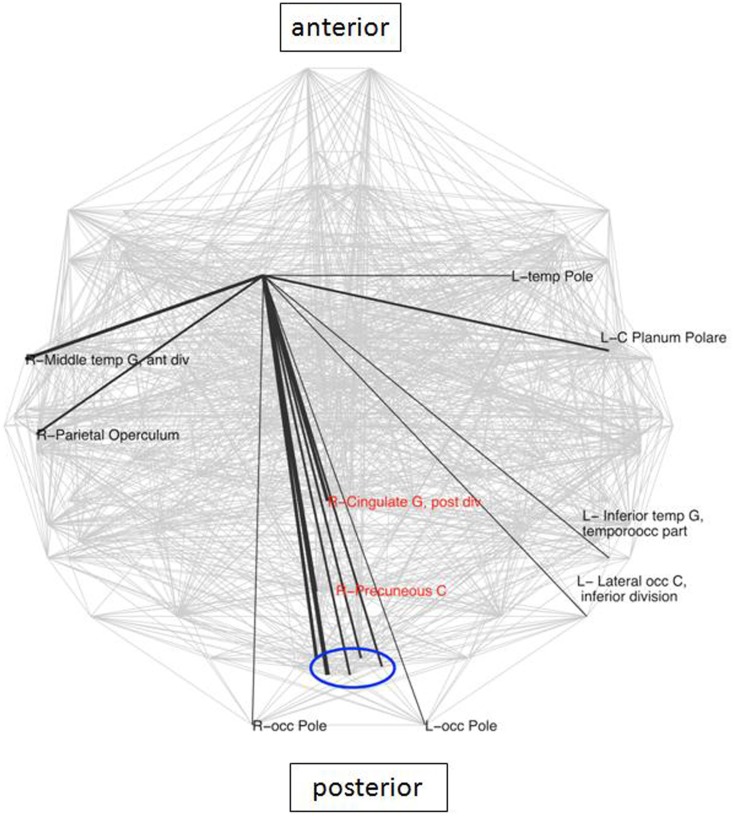
**Connections specific for the right caudate in LLD group**. These connections are superimposed on the whole brain connectivity (projected onto one axial plane), averaged for all subjects in each group (pale gray lines). The thickness of black lines is proportional to the frequency of occurrence of a particular caudate connection in relation to the total number of connections in each group. Depicted in red are core elements of the default mode network (DMN). R\L, right\left hemisphere; front, frontal; G, gyrus; inf, inferior; occ, occipital; blue oval depicts a cluster of closely located structures of the primary visual cortex, consisting of bilateral cuneal, and supracalcarine cortices as well as left lingual and intracalcarine cortices.

**Figure 4:**
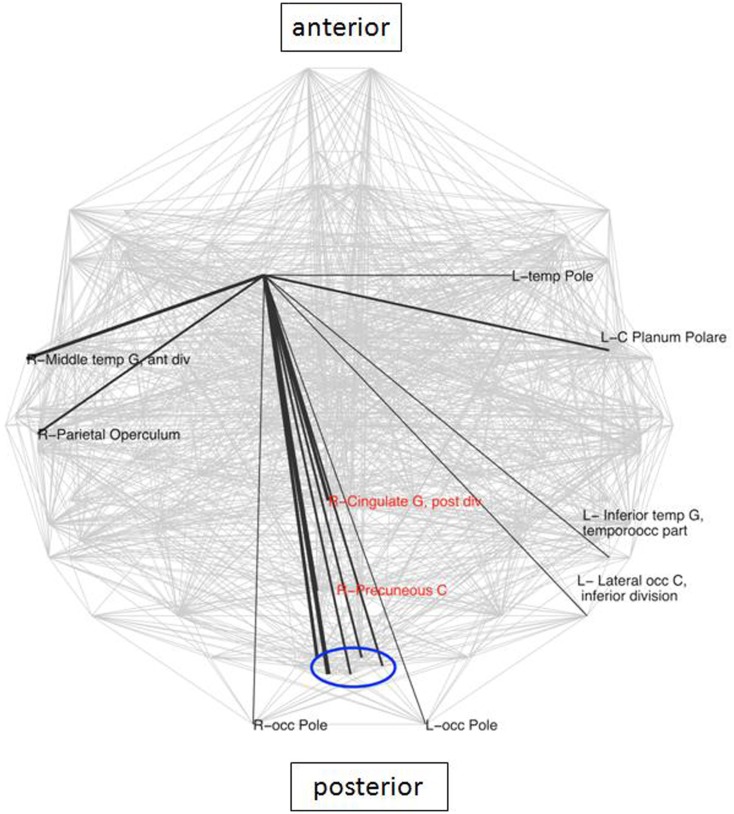
**Areas with significantly different frequencies of right caudate connections between the groups (*z*-score > 2) superimposed on the whole brain connectivity (projected onto one axial plane), averaged for all subjects in each group (pale gray lines)**. LLD-related increases in black, decreases in red (note: only connections shared in the two groups were taken into account), R\L, right\left hemisphere; G, gyrus; front, frontal; occ, occipital.

The caudate has been identified in earlier studies (Genovese et al., 2002) as a crucial area involved in LLD, due to its known role in emotion regulation. Analysis of connectivity of the right caudate in the present study between the groups demonstrated the existence of 16 nodes which were specific for the LLD subjects (Figure 3), importantly including the PCC and the PC, which are elements of the DMN. While looking at frequencies of neighbors of the right caudate nucleus (rCN) present in the two groups, six areas occurred more frequently in controls and three were more prevalent in LLD (*z*-score > 2 of pooled frequencies were considered as significantly different, see Figure 4). Despite variability in frequencies of hub’s occurrences between groups, connections with medial-parietal areas observed in the rCN tended to occur more frequently in LLD patients (Figure 3).

### Effect of antidepressant medications

A previous study (Anand et al., 2005b) reported an up-regulatory effect of selective serotonin reuptake inhibitors (SSRI) such as sertraline on connectivity between the anterior cingulate and limbic regions. To verify that our findings are not due to SSRI activity, we compared the global strength of connectivity (based on CM) and node-related average EDs for SSRI-takers (see Table 2) and patients not taking these drugs. We found no significant differences for both sub-groups. Based on these analyses, differences observed for controls vs. LLD group are unlikely to result from SSRI intake, although due to the small SSRI subgroup we cannot dismiss this effect entirely. Ideally, all depressed subjects would be medication free but the associated ethical concerns with this would be great.

## Discussion

In this study, we showed distinct differences in FC between LLD subjects and similarly aged healthy controls. First, at the global level, the average correlation strength is higher in LLD. Secondly, spatial properties of individual nodes were altered in LLD: 16 nodes showed a significant difference for average spatial (Euclidean) distance between connected nodes with 14 increased and two reduced distances (Figure 2). Third: the core hubs for LLD comprised the medial PCC and anterior supramarginal gyrus (ASG), whereas in controls the MFG was more common. Below we discuss these three major points.

### Depression: Increased or decreased connectivity?

Our analysis of strength of global association between nodes (of raw CM) yielded higher values for LLD. This is in agreement with a number of studies that have reported increased connectivity in depression. However, there have also been papers showing decreased connectivity in this condition. One possible explanation for this discrepancy might be significant methodological differences between studies. For example, some studies use model-free approaches (Greicius et al., 2007; Veer et al., 2010) whereas others use model-based approaches (Bluhm et al., 2009; Sheline et al., 2010; Zhou et al., 2010). FC can either be determined in the RS (Greicius et al., 2007; Bluhm et al., 2009; Sheline et al., 2010) or while performing tasks (Aizenstein et al., 2009; Grimm et al., 2009; Sheline et al., 2009). For studies with depression patients over 30 years of age, increased connectivity has generally been reported (Greicius et al., 2007; Bluhm et al., 2009) with few studies reporting decreased connectivity (e.g., Veer et al., 2010).

There are only few publications investigating FC in LLD (see e.g., Yuan et al., 2008; Aizenstein et al., 2009). A study reported decreased FC (Aizenstein et al., 2009) whereas the study by Zhang et al. (2011) in subjects with a wide range of age reported both increased (putamen, frontal, and parietal cortex) and decreased (frontal, temporal, and parietal cortices) FC. A recent study showed an increased global network integrity metrics based on graph theory (increased efficiency; decreased characteristic path length) and locally for a range of nodes (increased nodal centrality) in freshly diagnosed drug-naive patients (Zhang et al., 2011). The findings from the current study are partially consistent with reports of increased connectivity, at least as revealed at the level of raw cross-correlation matrices.

At nodal level tendencies toward increased connectivity was observed for all types of networks analyzed, but these differences did not survive FDR correction. Noteworthy one of the areas with a higher degree (number of neighbors) for binary graphs in LLD was the right anterior cingulate (31.93 ± 6.76 vs. 25.44 ± 7.55, *t*-test *p* = 0.018, uncorrected). In a previous study an increase of connectivity was reported in subgenual cortex, which is a small part of anterior cingulate (Greicius et al., 2007). Therefore it is tempting to hypothesize that an observed tendency toward increase in the number of connections for the anterior cingulate cortex was driven by the subgenual cortex. Psychosurgical interventions specifically target major projections and elements of the DMN such as anterior cingulate cortical tracts connecting it to other structures. In recent years, more refined methods include deep brain stimulation for treatment-resistant forms of depression. Interestingly, the subgenual cingulate cortex, one of the regions targeted by this technique for depression symptoms relief (Mayberg et al., 2005) was also found to be characterized by an increased FC in depression patients (Greicius et al., 2007).

### Late-life depression: Increase of correlation length

This is the first study to report increased average ED between many nodes in LLD (Figure 2). The increases in geometrical distances of average connections in LLD suggest the prevalence of long connections implying more intense communications between large and widely distributed components of the brain networks such as the DMN. Indeed, a recent study (Zhang et al., 2011) suggested that diminished average *L* in major depression is linked to an increased number of long-range connections. In addition, elements of the DMN were characterized by higher centrality metrics. An overall increase in long distance connections (observed in this study) could be explained by an up-regulation of DMN activity, as areas displaying higher ED values were core components of the posterior part of the DMN. Amongst the areas with up-regulated mean ED is the right caudate. This region also showed a more diffuse pattern of connectivity in a previous seed-based analysis of the same data (Sheline et al., 2009). The observed rise in ED may be regarded as another altered feature of connectivity related to the caudate associated with LLD.

### LLD and core hubs

fillskip0ptThe results of this study suggest that LLD spares general organization of FC, at least in relation to the aggregate topological measures used. This is in contrast to neurodegenerative diseases such as Alzheimer’s disease that show higher characteristic path lengths in FC and decrease in small-worldness properties (de Haan et al., 2009). In general, many neurodegenerative disorders seem to target specific elements of the brain that are considered to be critical parts of its topology (Buckner et al., 2009). We therefore specifically investigated the connectivity pattern and structure of the 15 top hubs (Table 3). Changes in composition of the core of hubs were observed, with LLD individuals having a higher frequency of medial PCC and one parietal structure ASG, whereas controls had a higher frequency in the MFG. Within the set of core hubs, a similar pattern was also determined by Kenny et al. (2010). PCC is a crucial component of the DMN and is thought to play a role in interpreting other people’s feelings and envisaging the future (Buckner et al., 2008). Importantly, it is part of the limbic system and disturbances in its connectivity, especially with

the frontal cortex, were related to psychiatric diseases including depression and schizophrenia (Buckner et al., 2008; Johnson et al., 2009). In line with connectivity abnormalities, a lower inhibition of DMN activity was shown in attention-demanding tasks in relation to depression (Anand et al., 2005a; Greicius et al., 2007; Auer, 2008; Buckner et al., 2008). Importantly a recent paper more specifically pointed to the significance of over-activity of the posterior medio-parietal complex comprising the PCC in major depression (Sheline et al., 2009). The role of SG in LLD is more difficult to interpret, however this structure lies in close proximity to parietal components of the DMN. The study by Buckner and co-workers identified SG as one of the critical cortical hubs, similarly in a DTI (Buckner et al., 2009), and morphometric connectivity study (Gong et al., 2009). Importantly, the authors also noted an overlap of the network comprising SG with a network containing PCC/PC (core constituents of the DMN) (Buckner et al., 2009).

### Experimental group composition and limitations of the study

The first possible concern about this study is the definition and composition of the patient group. Although this group was characterized by a spread in clinical characteristics (e.g., age of onset and number of depression episodes) the patients shared the features which were in the centre of our attention: the occurrence of depression in later life, rather than late-onset depression. Despite the variance in age of onset, all patients had suffered an initial episode of depression followed by remission with then at least another one episode in later life. Another point is that they were not depressed at the time the study was performed enabling us to look at patients state rather than trait. Therefore these findings may reflect features which are either a consequence of the previous disease or are constituent part of the brain organization in subjects vulnerable to depression. There is another possible concern. Although subjects were not currently depressed we did not include a specific measure of severity of anxiety symptoms and it is therefore possible that some of the changes in connectivity we identified reflected comorbid anxiety symptoms. Last but not least: there were medications taken by a part of patient group. Ideally, all depressed subjects would be medication free: however for a study looking for a long term effects of a disease, it is very difficult to recruit a sufficiently large group of patients completely free of medications. In addition we addressed a possible effect of SSRIs and found no significant impact on our findings.

It should be stressed that there are several potential confounds to the RS signal such as, for example, physiological noise (both respiratory and cardiac related). Over the last decade various approaches to remove potential noise have been assessed, but this still remains a key area of investigation (for reviews: Birn, 2012; Snyder and Raichle, 2012). In this study, we carried out high-pass temporal filtering and global signal removal to account for potential global noise in our data. More recently, other studies have regressed white matter and CSF signal and included motion parameters in their analysis. These methods are receiving growing attention and are being used (see e.g., Liao et al., 2010; Zuo et al., 2012) in addition to the methods that we used (e.g., Lynall et al., 2010; Sanz-Arigita et al., 2010).

In addition recent studies have applied corrections to the extracted BOLD signal to account for potential effects caused by brain atrophy (see e.g., Binnewijzend et al., 2012; Voets et al., 2012). Although atrophy is a potential confound, we did not observe significant differences in the brain volume between the groups, therefore we conclude that levels of atrophy might not be a factor that could explain FC differences between LLD patients and controls.

We believe that despite these limitations the study gives a valuable insight into the characteristics of the state of the brain affected by a relatively long history of a mental disease. It provides new information and/or corroborating previous findings or suggestions.

### Conclusion

This is the first FC study showing that in LLD specific brain areas are characterized by higher correlation lengths (EDs between nodes with correlated activity). In line with the above notion, the average functional correlation strength is higher in LLD. In contrast, clustering coefficient, characteristic path length, and modularity in tresholded binary networks were unaffected in LLD. In LLD, connectivity with the caudate nucleus (right) showed a more diffuse pattern and linked closer to the core elements of the DMN. In conclusion, this study reports some interesting findings of altered connectivity in LLD and highlights the potential use of RS FC in characterizing LLD.

## Conflict of Interest Statement

The authors declare that the research was conducted in the absence of any commercial or financial relationships that could be construed as a potential conflict of interest.

## Appendix

**Table A1:** **List of brain regions analyzed**.

Abbreviation	Brain region
FP	Frontal pole
IC	Insular cortex
SFG	Superior frontal gyrus
MFG	Medial frontal gyrus
IFG.pt	Inferior frontal gyrus, pars trigonum
PIFG.po	Inferior frontal gyrus, pars orbitale
PG	Precentral gyrus
TP	Temporal pole
STG.ad	Superior temporal gyrus, anterior division
STG.pd	Superior temporal gyrus, posterior division
AMTG	Medial temporal gyrus, anterior division
PMTG	Medial temporal gyrus, posterior division
MTG.top	Medial temporal gyrus, temperooccipital part
AITG	Inferior temporal gyrus, anterior division
TG	Temporal gyrus
ITG temp occ	Inferior temporal gyrus, temporal occipital part
PostcG	Postcentral gyrus
SPL	Superior parietal lobule
SGA	Supramarginal gyrus (SG), anterior division
PSG	Supramarginal gyrus, posterior division
AG	Angular gyrus
SLOC	Lateral orbital cortex, superior division
ILOC	Lateral orbital cortex, inferior division
ICC	Inferior calcarine cortex
FMC	Fronto medial cortex
SMC	Supplementary motor cortex
SCC	Subcalcarine cortex
PCG	Paracingulate gyrus
ACG	Cingulate gyrus, anterior division
PCG	Cingulate gyrus, posterior division
PC	Precuneal cortex
CC	Cuneal cortex
FOC	Frontal orbital cortex
APG	Parahippocampal gyrus, anterior division
PPG	Parahippocampal gyrus, posterior division
LG	Lingual gyrus
ATFC	Temporal fusiform cortex, anterior division
PTFC.	Temporal fusiform cortex, posterior division
TOFC	Temporo occipital fusiform cortex
OFG	Occipital fusiform cortex
FOpC	Frontal operculum cortex
COC	Central opercular cortex
POP	Parietal operculum cortex
PP	Planum polare
HG	Heschl’s gyrus
PT	Planum temporale
SCC	Supracalcarine cortex
OP	Occipital pole
Thal	Thalamus
NC	Nucleus caudatus
Put	Putamen
Pal	Pallidum
Amy	Amygdala
Acc	Nucleus accumbens
Hipp	Hippocampus

### Methods

#### Characteristic path length *L*

The shortest path metric may be applied to characterize the whole network or single nodes (brain regions) within it (Watts and Strogatz, 1998). The shortest path is defined as a path connecting two non-identical nodes *i* and *j* passing through a minimal number of edges. This number of edges is the length of the path. The average shortest path (characteristic path length, *L*) is defined as the sum of all shortest path lengths within the network divided by the number of all possible pathways between non-identical nodes in the graph (Eq. 1):

(A1) $$L=\frac{1}{N\times \left(N-1\right)}\overset{n}{\underset{i=l}{\sum }}\overset{n}{\underset{j=l}{\sum }}l_{ij};i\neq j$$

*N* – total number of nodes in the network

*l_ij_* – the shortest path between nodes *i* and *j*

for an average *L*:

(A1a) $$L_{\text{av}}=\frac{\overset{n}{\underset{i=1}{\sum }}L_{i}}{N}$$

In this study, Floyd’s algorithm was used (Floyd, 1962) to calculate *L*.

#### Clustering coefficient *C*

The clustering coefficient indicates how well neighbors of a node are connected (Watts and Strogatz, 1998). It specifies the proportion of edges within the neighborhood of a node *i* to the potential maximum number of edges between neighbors.

For a single node *i* the following formula was applied (Eq. 2)

(A2) $$C_{i}=\frac{N_{\text{c}}}{\left(N-1\right)\times N}$$

*C_i_* – clustering coefficient of node *i*

*N* – number of neighbors of node *i*

*N*_c_ – number of existing connections within the neighborhood of node *i*

The average clustering coefficient was subsequently calculated using the local clustering coefficient of each individual node, according to Eq. 3:

(A3) $$C_{\text{av}}=\frac{\overset{N}{\underset{i=1}{\sum }}i}{N}$$

*C*_av_ – average clustering coefficient

*C_i_* – clustering coefficient of each node in the graph

*N* – number of nodes in the network

#### Small-world index (σ)

The value of small-worldness is defined from values of *L* and CC comparing a tested network against a random benchmark network (having the same number of nodes and edges; Watts and Strogatz, 1998). The small-world index (σ; Humphries and Gurney, 2008) was then calculated according to Eq. 4:

(A4) $$\sigma =\frac{C_{\text{t}}\times L_{\text{r}}}{C_{\text{r}}\times L_{\text{t}}}$$

*C*_t_ – average clustering coefficient for a tested network

*C*_r_ – average clustering coefficient for a benchmark random network (result of averaging this metrics for six instances of a benchmark random network)

*L*_t_ – average shortest path length for a tested network

*L*_r_ – average shortest path length for a random network (result of averaging this metrics for six instances of a benchmark random network)

Formula (4) is an equivalent of the following expression:

(A5) σ=γ∕λ

where:

(A6) $$\gamma =C_{t}∕C_{r}\lambda =L_{t}∕L_{r}$$

for networks with SW properties γ is typically above 2 and λ ∼ 1 (Humphries et al., 2006).

Measures for six instances of the random benchmark network were averaged for each subject. The same values of random benchmark network as for the whole network were used for node-wise analysis.

#### Modularity (*Q*)

Modules may be defined as communities of nodes within a graph which are more densely connected to each other than to the rest of the network (Clauset et al., 2004).

To estimate modular organization of networks, a fast modularity maximization algorithm was applied (Clauset et al., 2004). Adjacency matrix representations of FC networks were converted into their incidence versions using a Java programme employing the JMatIO library (Gradkowski; http://www.mathworks.co.uk/matlabcentral/fileexchange/10759) which were then input to a fast modularity algorithm (http://www.cs.unm.edu/aaron/research/fastmodularity.htm) to calculate modularity, *Q*.

Maximal modularity *Q* was treated as the modularity for each network. Modularity (*Q*) is a reflection of the natural segregation within a network (Newman, 2004) and can be a valuable tool in identifying the functional blocks within. Given two parcellations into distinct modules for the same network, the parcellation with the higher value of *Q* would be preferred. Note that *Q* does not include information about how many modules exist or about their size or overlap.

### Discussion

#### Variability in sub-circuit properties

In contrast to aggregate topology metrics, all values for microcircuits (the top 15 hubs and BG) were characterized by remarkable inter-individual variability. This seems to reflect a general feature of RS FC. Honey et al. (2009) looked into this problem and found a poor correlation (with r ranging from 0.4 to 0.6) not only between individuals but also within the same subjects when scanned on two different sessions. This variability may be due to the fact that although there is a common denominator to the brain activity in RS, there are also a plethora of differences. A likely cause is that subjects may engage in a variety of types of mental activity during a RS study. In fact, in the light of Buckner et al. (2009), RS may be seen as an umbrella term covering different types of internal cognitive processes, such as theory of mind, autobiographical memory, planning, and others. There are also a number of methodological issues that probably contribute to the observed variability. FC may be determined using many approaches. The most popular of them (also used in this study) is based on generating thresholded, binary cross-correlation matrices (un-directed, unweighted graphs). There are also other means of representing functional brain networks, including: partial correlation, mutual information, or synchronization likelihood (summarized in Bullmore and Bassett, 2010). In addition, pre-processing techniques, such as filtering and treatment of the global signal, may also play an important role (Fox and Raichle, 2007; Weissenbacher et al., 2009). Although in the term FC, the word connectivity is used, we should bear in mind that it deals with merely temporal coherence in BOLD between different, distributed brain areas. This coherence in some cases does not reflect the existence of direct activation of one by the other (Honey et al., 2009) and should therefore not be confused with effective connectivity (Sporns et al., 2004). In fact there may be different connectivity patterns resulting in coherence in BOLD detected for a pair of nodes. Major possible causes include (a) intermediate nodes, resulting in coherence between two nodes, even though there is no direct connection between them and common input, such as activity of neuromodulatory ganglia activating distributed regions of the neocortex, e.g., cholinergic transmission originating in BG or other “synchronizers” such as vast neuromodulatory transmission from brainstem or unspecific thalamic nuclei.

Due to these and other reasons, structural connectivity is considered to yield results in greater agreement with anatomy (Iturria-Medina et al., 2007). Therefore some authors postulate using structural connectivity as guidance for FC (Rykhlevskaia et al., 2008; Honey et al., 2009).

In a broad sense RS FC has been proven to be a useful tool to determine changes in global brain organization in relation to diseases such as AD or other dementias, affecting critical components, and global properties of brain networks (Stam et al., 2007; Buckner et al., 2009; de Haan et al., 2009; Seeley et al., 2009). In other brain impairments, for example depression, this method may have shortcomings mostly due to unaffected or weakly affected large-scale organization of FC.
